# An updated review summarizing the anticancer potential of flavonoids via targeting NF-kB pathway

**DOI:** 10.3389/fphar.2024.1513422

**Published:** 2025-01-06

**Authors:** Pratibha Pandey, Sorabh Lakhanpal, Danish Mahmood, Han Na Kang, Byunggyu Kim, Sojin Kang, Jinwon Choi, Min Choi, Shivam Pandey, Mahakshit Bhat, Shilpa Sharma, Fahad Khan, Moon Nyeo Park, Bonglee Kim

**Affiliations:** ^1^ Centre for Research Impact and Outcome, Chitkara University Institute of Engineering and Technology, Chitkara University, Rajpura, Punjab, India; ^2^ School of Pharmaceutical Sciences, Lovely Professional University, Phagwara, Punjab, India; ^3^ Department of Pharmacology and Toxicology, College of Pharmacy, Qassim University, Buraydah, Saudi Arabia; ^4^ KM Convergence Research Division, Korea Institute of Oriental Medicine, Daejeon, Republic of Korea; ^5^ Department of Pathology, College of Korean Medicine, Kyung Hee University, Seoul, Republic of Korea; ^6^ School of Applied and Life Sciences, Uttaranchal University, Dehradun, India; ^7^ Department of Medicine, National Institute of Medical Sciences, NIMS University Rajasthan, Jaipur, India; ^8^ Chandigarh Pharmacy College, Chandigarh Group of Colleges-Jhanjeri, Mohali, Punjab, India; ^9^ Center for Global Health Research, Saveetha Medical College, Saveetha Institute of Medical and Technical Sciences, Chennai, India

**Keywords:** flavonoids, natural compounds, NF-kB, signaling/signaling pathways, anticancer

## Abstract

Nuclear factor-κB (NF-κB) cell signaling pathway is essential for the progression and development of numerous human disorders, including cancer. NF-κB signaling pathway regulates a wide range of physiological processes, such as cell survival, growth, and migration. Deregulated NF-kB signaling resulted in unregulated cell proliferation, viability, movement, and invasion, thus promoting tumor development. Recent findings have increasingly shown that plant derived phytochemicals that inhibit NF-κB signaling have the potential to be employed in cancer therapeutics. Flavonoids are a group of polyphenolic natural compounds present in various plants and their fruits, vegetables, and leaves. These compounds have numerous medicinal properties owing to their antioxidant, anti-inflammatory, antiviral, and antitumor characteristics. The main mechanism by which these flavonoids exhibit their anticancer potential is via potent antioxidative and immunomodulatory actions. Current research reports have demonstrated that these flavonoids exhibited their anticancer effects via suppressing the NF-κB signaling. Based on these facts, we have comprehensively outlined the cancer promoting role of NF-κB pathway in various processes including tumor progression, drug resistance, angiogenesis and metastasis. In addition to these, we also summarize the anticancer potential of flavonoids by specifically targeting the NF-κB pathway in various types of cancers.

## Introduction

**FIGURE 1:**
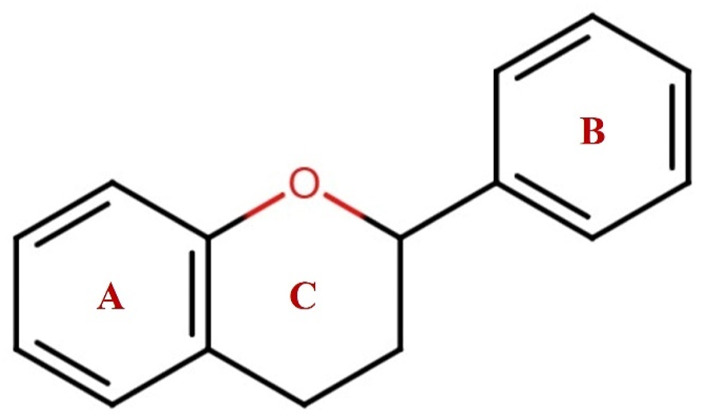
The general structure of flavonoids containing C6-C3-C6 has two benzene rings, A and B, connected with a heterocycle pyrene ring C.

Medicinal plants have long been recognized as rich sources of natural compounds with immense therapeutic potential. Plant-derived secondary metabolites are commonly utilized in the treatment and prevention of various chronic diseases, either on their own or in conjunction with other medications (Tungmunnithum et al., 2018). Phytochemicals are categorized into various groups, including phenols, alkaloids, flavonoids, anthraquinones, glycosides, glucosinolates, and organosulfur compounds. Phenolic compounds such as flavonoids, phenolic acids, tannins, stilbenes and coumarins, are frequently present in dietary and medicinal plants (Yuan et al., 2022; Oyenihi and Smith, 2019). Flavonoids, the most common phenolic compounds, typically decrease the probability of chronic illness by demonstrating their antioxidant potential. They are classified into flavonols and flavones. Phytochemicals are known for balancing oxidative stress caused by reactive oxygen species (ROS) in the body, leading to therapeutic benefits (Jakobušić Brala et al., 2023). Flavonoids are a group of natural compounds found in various plants, fruits, grains, flowers, and beverages such as tea and wine (Sun and Shahrajabian, 2023). A healthy diet is universally acknowledged as essential for the prevention or amelioration of numerous diseases, including cancer and its progression, cardiovascular and neurodegenerative diseases (Aiello et al., 2021; Barreca et al., 2023). Thus, these substances are recognized as dietary flavonoids. Flavonoids are classified into various subclasses based on the connection of the C ring to the B ring and on the degree of unsaturation and oxidation of the C ring (Figure 1) (Shen et al., 2022). Based on the variation of chemical structure, we can differentiate between the subgroups of flavonoids such as isoflavones, neoflavonoids, flavones, flavanones, flavonols, flavanols, flavanonols, chalcones, and anthocyanins. Certain natural phytocompounds possess specific subgroups of compounds. For instance, tea and onions are rich dietary sources of various classes of flavonoids. Leguminous species are the primary sources of isoflavones. Proanthocyanidins can be present in the seeds, flowers, and fruits of various plant groups like apples, grapes, blueberries, and certain cereals. Red wine is rich in flavanols different types of flavonols (like epicatechin myricetin and quercetin), anthocyanins (malvidin-3-glucoside), and resveratrol, which are renowned for their antioxidant properties (Drețcanu et al., 2022; Bešlo et al., 2023).

**FIGURE 2:**
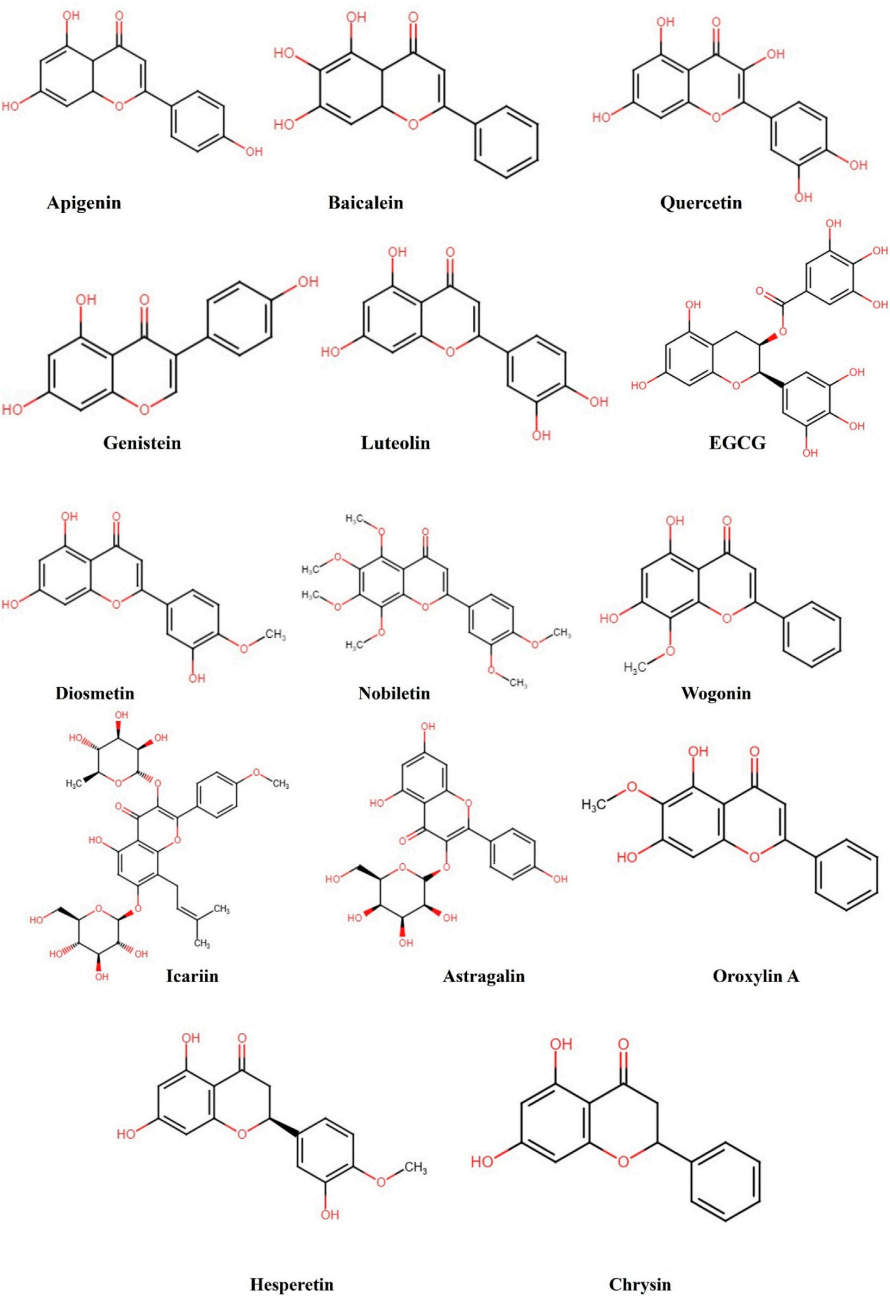
Structural representation of the chemical formula of flavonoids targeting NF-κB pathway in cancer.

Typically, conventional medicinal products have been used to treat a range of inflammatory disorders and cancers. Cancer is a complex disorder that can be influenced by a variety of genetic, epigenetic, environmental, and hormonal factors. A wide range of signaling pathways are triggered in various types of cancer (Sanchez-Vega et al., 2018). Cell signaling pathways in cancer cells, including the PI3K/Akt/mTOR, NF-κB, JAK/STAT, MAPK/ERK, Notch, Wnt, and TGF-β pathways, play a crucial role in drug resistance and metastasis (McCubrey et al., 2015). Phytochemicals have the potential to influence cancer cells by targeting specific signaling pathways. It is evident from this observation that phytochemicals have a significant impact on NF-κB signaling, which plays a crucial role in the development of inflammatory diseases and cancer. Polyphenols, such as flavonoids, are known for their remarkable anti-inflammatory and antioxidant attributes as well as their ability to influence molecular targets and signaling pathways, which contribute to their effectiveness in fighting cancer (Chen and Liu, 2018). NF-κB plays a crucial role as a nuclear transcription factor in cellular inflammation and immune response. Given the significant impact of NF-κB on inflammation, immunity, and cancer, extensive research has demonstrated its strong association with cancer cell survival, drug resistance, angiogenesis and metastasis. It is evident that NF-κB plays a promising role in the development and advancement of cancer. Thus, NF-kB holds significant value as a molecular target in cancer research (Jiang et al., 2023). Consequently, considerable attention has been targeted on the identification and development of drugs that specifically target NF-κB pathway in order to address various forms of cancer. NF-κB presents itself as a promising therapeutic target for pharmaceutical interventions. Natural products containing flavonoid components offer special benefits for tumors. It has the ability to exert the antioxidant and anti-inflammatory properties by inhibiting NF-κB activity, which ultimately leads to anti-tumor effects (Das et al., 2022). This review attempts to investigate the role of NF-κB in angiogenesis, metastasis, carcinogenesis, and proliferation. It also evaluates the state of the art in terms of research on the various anticancer effects of flavonoid compounds through modulating the NF-κB pathway (Figure 2).

## Role of NF-κB signaling in carcinogenesis

Various stimuli can trigger activation of the NF-κB signaling pathway during both normal and abnormal events. It has been reported that NF-κB plays a crucial role in the process of tumorigenesis (Keerthy et al., 2014). During cancer progression, activation of the NF-κB signaling pathway can stimulate various characteristics of tumor cells, such as proliferation, angiogenesis, and invasion (Wang et al., 2020; Gupta et al., 2023; Shi et al., 2023). Certain substances have been found to be capable of inhibiting NF-κB signaling and thus have the potential to prevent the development of cancer (Ramadass et al., 2020). For example, when astaxanthin is loaded onto solid lipid nanoparticles, it can lead to downregulation of NF-κB signaling, which in turn inhibits breast cancer (Sun et al., 2019a). The process of inflammation plays a critical role in augmenting cancer cell growth and survival of cancer cells. When NF-κB is activated, it triggers inflammation that can accelerate the progression of cancer (Yuan et al., 2024). Citronellol functions as an antitumor agent by decreasing TNF-α, IL-6, and inflammation, which in turn downregulates NF-κB expression. This was achieved by effectively reducing the expression of inflammatory markers such as TNF-α, IL-6. This approach is intriguing because of its potential to block the development of breast cancer (Jayaganesh et al., 2020). The interaction between TNF-α and NF-κB in cancer is quite complex. It is clear that TNF-α plays a crucial role in the activation of NF-κB signaling. Studies have shown that TNF-α can trigger the movement of RelA into the nucleus, which in turn promotes the spread of cancer cells and aids their transition into a more aggressive form (Zhao et al., 2020). Helichrysetin, an anti-tumor agent, has the ability to effectively suppress cancer progression by preventing TNF-α-mediated NF-κB signaling (Wang et al., 2021a). Notably, NF-κB can enhance IL-6 levels in cancer cells. IL-6 triggers the production of STAT3, a factor that promotes tumor growth in the process of carcinogenesis (Zhang et al., 2021). Interestingly, STAT3 could also function as an upstream regulator of NF-κB signaling in cancer cells. Studies have shown that celecoxib, when used as an antitumor agent, can reduce tumor differentiation and metastasis by suppressing the STAT3/NF-κB axis (Zuo et al., 2018). Because of the probable role that this axis plays in facilitating immune evasion of cancer cells, targeting the NF-κB signaling pathway is an ideal method in the field of cancer immunotherapy. PD-L1 expression is known to increase in cancer cells that develop resistance to immunotherapy. PD-L1 has the ability to reduce the inhibitory potential of lymphocytes against cancer cells and repress the cancer cell growth (Kong et al., 2020a; Setordzi et al., 2021). It has been observed that the activation of NF-κB can lead to the upregulation of PD-L1 in the tumor microenvironment, which in turn stimulates the proliferation of cervical carcinoma cells (Cai et al., 2020). Research has shown a strong link between NF-κB signaling and the advancement of cancer, as well as a negative outcome in patients with cancer (Quinn et al., 2020). Through the study of molecular mechanisms, it has been observed that the NF-κB signaling pathway plays a promising role in the survival of cancer cells. When stimulated, NF-κB has the ability to shield thyroid cancer cells from apoptosis and promote G1/S cell cycle progression (Faria et al., 2017). Downregulation of NF-κB can result in the upregulation of caspase-3 and induction of apoptosis in ovarian cancer cells. Moreover, the inhibition of NF-κB signaling has been linked to decreased levels of anti-apoptotic protein, Bcl-2, and matrix metalloproteinase-9 (MMP-9), which plays a role in cancer metastasis (Yang et al., 2018). The significance of NF-κB signaling stems from its involvement in chemoresistance. Given the ongoing battle against drug resistance in cancer therapy, there has been significant interest in exploring the downregulation of NF-κB signaling as a potential solution to reversing chemoresistance (Manu et al., 2015; Ahmed et al., 2024). In ovarian cancer cells, c-Myb plays a crucial role in promoting NF-κB signaling, which in turn contributes to cancer growth, invasion, and resistance to cisplatin chemotherapy (Tian et al., 2019). Notably, NF-κB also plays a role in promoting cancer growth by triggering glycolysis (Warburg effect) (Ye et al., 2018). It seems that the phosphorylation of RelA can also contribute to glycolysis and promote cancer progression alongside glucose transporter 1 (GLUT1) (Tian et al., 2021). In addition, certain factors, such as protein arginine methyltransferase 5 (PRMT5), can activate NF-κB signaling, which in turn enhances glycolysis in cancer cells (Han et al., 2020). In addition, there have been reports indicating that the NF-κB signaling pathway can hinder the expression of factors that suppress tumor growth. As an example, phosphatase and tensin homolog (PTEN) has the ability to repress the proliferation of cancer cells by suppressing the PI3K/Akt axis (Gehringer et al., 2020; Hashemi et al., 2023; Alimohammadi et al., 2024). Consequently, it has been found that NF-κB activation potentially enhance cancer cell proliferation and viability by inhibiting PTEN (Man et al., 2019). In general, research supports the idea that NF-κB largely acts as a pro-carcinogenesis factor in various types of cancer, and modulating its expression could potentially be a useful approach for cancer treatment (Pakjoo et al., 2024; Ebrahimi et al., 2024).

## Flavonoids as NF-κB inhibitors

Medicinal plants have been an important source of diverse anticancer medications used in chemotherapy. Phytochemicals, also referred to as bioactive compounds, have been found to be a potent candidate in the treatment of cancer (Situmorang et al., 2024). These sources are reliable, safe, cost-effective, and readily available, covering a wide range of locations from rural to urban areas through various nations (Yuan et al., 2022). Thus, there is an increasing interest in investigating the potential anticancer properties and mode of action of phytocompounds. Chemoprevention involves the utilization of different natural and synthetic chemicals to suppress or inhibit the cancer progression by targeting specific molecular signaling pathways (Ranjan et al., 2019). Currently, scientific research is focused on determining the underlying mechanism of phytochemicals on cellular signaling. These compounds exhibit unique mechanisms of action in combating tumors (Abutayeh et al., 2024). Numerous biological mechanisms and signaling molecules influences the gynogenesis movement. Signaling pathways and molecular networks are essential for regulating important cellular activities required for cell growth and survival. It is necessary to correlate the etiology of cancer with different signaling pathways that are biologically important (Fawaz et al., 2023). Multiple molecular biology approaches have been devised for the detection and treatment of cancer. These include directing treatment towards cancer stem cell pathways, using retroviral therapy, suppressing oncogenes, and modifying tumor suppressor genes (Hermayerni Simanullang et al., 2022). Different cell signaling pathways, such as PI3K, Akt, mTOR, MAPK/ERK, Wnt, Notch, and Hedgehog, are linked with the control of cancer cell proliferation, invasion, migration, angiogenesis, and metastasis (Lee et al., 2024). Phytochemicals are responsible for the regulation of specific pathways or components, which in turn leads to the induction of anticancer effects.

**FIGURE 3:**
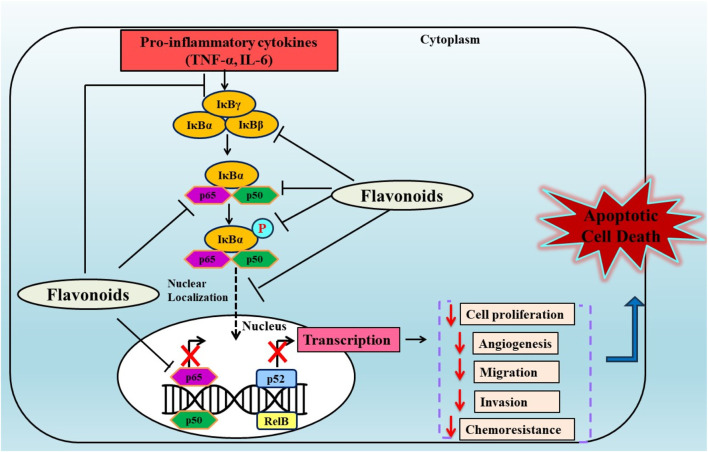
Anticancer effects of plant derived flavonoids by modulating the NF-κB pathway. Flavonoids exerts their effects by inhibiting the phosphorylation of NF-κB subunits (p65, p50) and downregulating the expression of NF-κB in various carcinomas.

Numerous independent research findings have demonstrated that various phytochemicals have anticancer effects via targeting different signaling pathways (Grover et al., 2024). However, there is a lack of specific information of the natural compound composition of plant constituents including, flavonoids, alkaloids, phenols, saponins, and terpenoids in context to their potential as antitumor agents (Almilaibary, 2024). Plant-based compounds have demonstrated significant efficacy in fighting cancer in recent years, offering clear benefits of being very efficient and having minimal toxicity. These compounds exhibited a critical role in regulating different processes in cancer cells, such as programmed cell death, migration, and senescence-related signaling pathways. This is achieved by controlling various important process and markers such as ROS level, MAPK pathway, Notch pathway, NF-κB pathway, and glycolytic enzymes (Hashem et al., 2022; Pandey et al., 2023a). Flavonoids are recognized as the most significant bioactive natural compounds and the most widely spread dietary phytochemicals with numerous pharmacological and biological characteristics (Kandaswami et al., 2005). Flavonoids have been found in both clinical and epidemiological investigations to have the ability to stop cancer in its tracks, both from starting (cytoprotection) and from spreading to other parts of the body (cytotoxicity) (Hazafa et al., 2020). Furthermore, they exert a suppressive effect on various types of malignancies including lung, bladder, breast, cervical, gastrointestinal, and others (Niedzwiecki et al., 2016). Multiple studies have documented that numerous flavonoids control the expression of NF-κB and the modification of chromatin by activating or inhibiting enzymes that are associated to epigenetic processes, such as histone acetyltransferases (HATs), histone deacetylases (HDACs), and DNA methyltransferases (DNMTs) (Selvakumar et al., 2020). This review explored various flavonoid phytochemicals, including their structures and potential mechanisms (Figure 3), by targeting NF-κB pathway to combat cancer (Table 1). With its extensive coverage, it provides a wealth of information on the potential of natural compounds specifically flavonoids in fighting cancer.

**TABLE 1:** Preclinical evidence of NF-κB mediated anticancer effects of flavonoids in various cancers.

Compound	Cancer	Model	Target	Mechanism	References
Apigenin	Breast cancer	MCF-7 cells	Repressed the phosphorylation level of IκBα	Induced extrinsic apoptosis pathway	Seo et al. (2012)
	Prostate cancer	PC-3 and 22Rv1 cells; xenograft mouse model	Inhibition of p-IKKα, NF-κB/p65	Lowers proliferation and enhances apoptosis	Shukla et al. (2015a)
	Prostate cancer	TRAMP mice	Blocked phosphorylation and degradation of IκBα, NF-κB	Decreased tumor size	Shukla et al. (2015b)
	Malignant mesothelioma	C57BL/6 mice	Inhibited NF-κB nuclear translocation	Decreased tumor size	Masuelli et al. (2017)
	Non-small cell lung cancer	A549 and H1299; A549 cells induced xenograft mice	Suppressed NF-κB	Induced apoptosis, suppressed tumor growth	Chen et al. (2016)
Baicalein	Breast cancer	MCF-7 and MDA-MB-231 Cells xenograft in female BALB/c nude mice	↓NF-κB, and p-IκB, ↑Manifestation of IκB at the protein level	Induced apoptotic cell demise and autophagy, reduced cell progression and metastasis	Yan et al. (2018)
	Oral cancer	Human oral Cancer SCC25 cells heterograft in BALB/c nude lab rat	↓NF-κB ↓p65 and p50	Suppressed tumor growth and induced apoptosis	Gao et al. (2020)
	Colorectal cancer	Colorectal cancer cell xenograft mouse model	↓NFκB phosphorylation	Suppressed tumor growth, metastasis and angiogenesis	Chen et al. (2021)
	Lung cancer	A549 cells induced Male BALB/c-nu mice	↓NFκB	Anti-inflammatory response induced apoptosis	Yu et al. (2017)
Quercetin	Oral squamous cell carcinoma	SAS cell line	↓NF-κB p65, IKB-α/β	Repression of migration and invasion	Lai et al. (2013)
	Liver cancer	HepG2	↓NF-κB	Anti-inflammatory and antigenotoxicity effects	Ramyaa et al. (2014)
	Colon cancer	CACO-2 and SW-620 cells	↓NF-κB DNA binding activity	Induced apoptosis	Zhang et al. (2015a)
	Prostate cancer	LNCaP,DU-145 and PC-3	↓pNF-κB	Antiproliferative effects	Ward et al. (2018)
	Colon, liver, breast and prostate cancer	HCT-116, HepG2, MCF-7, and PC3 cell	↓NF-κB	Apoptosis induction	Sahyon et al. (2022)
Genistein	Breast cancer	MDA-MB-231	↓NF-κB	↓Cell growth, apoptosis induction and cell cycle arrest	Pan et al. (2012)
	Prostate cancer	LNCaP, PC-3 cells	NF-κB inhibition	↓Cell growth, apoptosis induction	Davis et al. (1999)
	Bladder cancer	253J B-V	Downregulation of NF-κB	↓Cell growth, and cell cycle arrest	Singh et al. (2006)
	Multiple myeloma	U266	NF-κB inhibition	↓Cell growth, apoptosis induction	Xie et al. (2016)
	Thyroid cancer	CAL-62, ACC 448	Downregulation of NF-κB	↓Cell viability	Ozturk et al. (2018)
Luteolin	Breast cancer	MCF-7	Downregulation of NF-κB	Repression of migration and invasion	Park et al. (2013)
	Breast cancer	MDA-MB-231	↓pNF-κB	↓Cell proliferation	Huang et al. (2019)
	Pancreatic cancer	BxPC-3 human pancreatic cancer cell athymic nude mice	Downregulation of NF-κB p65	↓Cell proliferation	Johnson et al. (2015)
	Lung cancer	A549 cells	Suppressed IκB-α expression	Repression of migration and invasion	Chen et al. (2013b)
	Hepatocarcinoma	HepG2	NF-κB inhibition	↓Cell proliferation and release of reactive oxygen species (ROS)	Hwang et al. (2011)
EGCG	Breast cancer	MDA-MB-231	Downregulation of NF-κB	Repression of invasion	Sen et al. (2010)
	Ovarian cancer	ES2 and OVCAR3 cell line	Downregulation of NF-κB	↓Cell proliferation and invasion	Tian et al. (2019)
	Bladder cancer	SW780 cells	Downregulation of NF-κB	↓Cell growth, apoptosis induction and suppressed migration	Luo et al. (2017)
	Breast cancer	MCF-7, MDA-MB-231; E0771 cells injected C57BL/6J female mice	Inhibited NF-κB activation	Inhibiting tumor growth, proliferation, migration, and angiogenesis	Gu et al. (2013)
	Hepatocellular carcinoma	HCCLM6 cells	Downregulation of NF-κB	Induced apoptosis and cell cycle arrest	Zhang et al. (2015b)
	Pancreatic cancer	MIAPaCa-2 and SU.86.86 cells	Inhibited p65-NF-κB activity	↓Cell growth, apoptosis induction	Suhail et al. (2023)
Diosmetin	Colon cancer	HCT-116, HT-29	Inhibited NF-κB translocation	Inducted apoptosis and inhibited cell proliferation	Koosha et al. (2019)
	Hepatocellular carcinoma	HepG2	Downregulation of IKKα/β and NF-κB	Inhibited cell viability and stimulates cell apoptosis	Qiao et al. (2016)
	Gastric cancer	AGS cells	↓pNF-κB	↓Cell proliferation and invasion	Zhang and Luo (2023)
Nobiletin	Breast cancer	MDA-MB-231 cells	Suppressed activation of NF-κB	Repressed tumor cell metastasis and invasion	Baek et al. (2012)
	Gastric cancer	AGS cells	Downregulation of NF- κB (p65 and p50)	Repression of migration and invasion	Lee et al. (2011)
	Osteosarcoma	U2OS and HOS cells	Reduced the phosphorylation of p-IKKα/β and downregulation of NF- κB	Reduced cells motility, migration and invasion	Cheng et al. (2016)
	Prostate cancer	PC-3 and DU-145 cells	Downregulation of NF- κB (p50)	Suppressed cell viability	Chen et al. (2014)
	Ovarian cancer	OVCAR-3 and A2780/CP70 cells	Downregulation of NF- κB (p50)	↓Cell proliferation and angiogenesis	Chen et al. (2015)
	Pancreatic cancer	MIAPaCa-2	Downregulation of NF- κB	Inhibited the growth and metastasis	Jiang H. et al. (2020)
	Breast cancer	MCF-7 and BT-549	Suppressed level of IκB	Inhibited cell viability and stimulates apoptosis, pyroptosis	Wang et al. (2021b)
	Breast cancer	MDAMB-231 cells	Suppressed activation of NF-κB	↓Cell proliferation	Kim et al. (2022)
Wogonin	Hepatocellular carcinoma	Bel7402 and HepG2 cells	Suppressed phosphorylation of IκB and p65	Suppressed the proliferation and invasion	Liu et al. (2016)
Icariin	Cervical cancer	U14 and SiHa cells; U14 tumor-bearing mice	Downregulation of NF-κB p65	Inhibited tumor growth, proliferation, migration, angiogenesis and induced apoptosis	Li et al. (2021)
	Breast cancer	MDA-MB-231, MDA-MB-453, 4T1 cells; 4T1 or MDA-MB-231 mouse xenograft	Downregulation of NF-κB p65	Induced apoptosis and inhibited the migration	Song et al. (2020)
	Colorectal Cancer	HCT116 and HT29; HCT 116 cells athymic BALB/c mice	Downregulation of NF-κB	Inducted apoptosis and inhibited cell proliferation	Zhang et al. (2014)
	Colorectal Cancer	HT29 and HCT116; HCT116 cells nude mice xenograft	Downregulation of NF-κB	Suppressed tumour growth and chemoresistance	Shi et al. (2014)
	Hepatocellular carcinoma	SMMC-7721 and HepG2; HepG2 xenograft mice	Downregulation of NF-κB	Inhibited proliferation and induced apoptosis	Li et al. (2014)
Astragalin	Gastric cancer	AGS cells	Downregulation of NF-κB	↓Cell proliferation	Radziejewska et al. (2022)
	Colorectal cancer	HCT116 cells	Inhibited the NF-κB P65	Inhibited the proliferation and migration	Yang et al. (2021)
	Lung cancer	A549 and H1299	Inhibited NF-κB activation	Suppressed tumor growth and induced cancer cell apoptosis	Chen et al. (2017)
Oroxylin A	Breast cancer	MDA-MB-231	↓NF-κB	↓Cell proliferation, invasion, migration, EMT, ↑G1 arrest	Sun et al. (2019b)
	Colon cancer	HCT-116	↓NF-κB	↓Cell proliferation	Yao et al. (2014)
	Acute myelogenous leukemia	HL-60, NB4	↓NF-κB	↑TNFα sensitivity and growth inhibition	Li et al. (2018)
	Lung cancer	H460	↓NF-κB	↓Cell growth	Shen et al. (2017)
	Lung cancer	EA.hy926	↓NF-κB	↓Angiogenesis	Zhao et al. (2018)
Hesperetin	Glioblastoma	GL261 cells	↓NF-κB	↓Cell proliferation and metastasis, ↑ apoptosis	Cheng et al. (2022)
	Lung cancer	A549 and A549/DDP cells; A549/DDP cells xenograft mice	Inhibited the NF-κB P65 activity and translocation	↑ cisplatin sensitivity, inhibited tumor growth	Kong et al. (2020b)
Chrysin	Lung cancer	A549 and H1975 cells	Suppressed phosphorylation of IκB, IKKβ and nuclear level of p65	↓Cell proliferation, invasion, migration	Wu et al. (2018)
	Lung cancer	Benzo(a)pyrene induced lung cancer mouse model	↓NF-κB	Decreased tumor growth	Kasala et al. (2016)

## Apigenin

Apigenin is a flavonoid class phytocompound that can be obtained in large quantities from fruits, vegetables, herbs, and plant-based beverages. Several studies have reported the antitumor the potential of apigenin against different kinds of human carcinomas, including breast, cervical prostate, ovarian, lung, gastrointestinal cancers, melanoma, glioblastoma, osteosarcoma, and leukemia (Ghițu et al., 2019; Imran et al., 2020; Kashyap et al., 2018). This is achieved by reducing cell migration, promoting cell apoptosis, and boosting the immune response of cells, resulting in a decrease in the motility rate of cancer cells (Pandey et al., 2023b).

Apigenin’s anticancer efficacy is mediated by suppression of expression level of iNOS, COX-2, cyclin D1, and MMP-9 genes. All of these genes are regulated by the NF-kB pathway. Apigenin has been found to induce apoptosis in various types of cancer by inhibiting DNA replication and protein kinase activity, generating reactive oxygen species (ROS), causing damage to mitochondria, and disrupting the interaction between Ku70 and Bax (Naponelli et al., 2024). Cell proliferation and survival are linked to the NF-κB pathway, which triggers transcription of specific genes and their products. These products have the potential to hinder various stages of programmed cell death (Amini et al., 2023). This pathway consists of various dimers formed by different segments, including p50, p52, p65, RelB, and c-Rel proteins. The Investigator’s Brochure (IB) family consists of inhibitors such as p100, p105, IB, IκB, and Bcl-3 which play a crucial role in regulating NF-κB proteins. NF-κB heterodimers containing p65 and p50 are present in the cytoplasm and associate with IB subunits in their inactive state. By phosphorylating the IB subunit on a serine residue, activation of the kinase complex (IKK) triggers ubiquitination and proteasomal degradation, leading to NF-κB transcriptional activation and nuclear localization (Shukla et al., 2014a). Abnormal NF-κB activation has been linked to hematological cancers and cancers of the uterine cervix, lung, prostate, and pancreas (Shukla et al., 2014b). The researchers observed that NF-κB is consistently activated in tissue samples of prostate cancer, xenografts model, and in the TRAMP mice, which replicates many forms of advanced human prostate cancer (Karin, 2006). Increased NF-κB activity is associated with the development of various diseases, while its nuclear localization is correlated with a negative prognosis and higher likelihood of recurrence (Chaturvedi et al., 2011). Furthermore, the various family members of IB protein such as p100, p105, IκB, and Bcl-3 regulate the function of crucial mediators of NF-κB pathway. NF-κB is found in a heterodimeric state within the cytoplasm and forms an inactive state when it binds to IB. Several signaling molecules, including FasL, TNF-α, and TRAIL, can activate the IKK complex, leading to IB phosphorylation and degradation. NF-κB subsequently translocated inside the nucleus and activated their target genes which are involved in cellular survival, cell cycle, inflammation and tumor progression (Shukla et al., 2004). Apigenin therapy has been found to effectively prevents NF-κB activation in a wide range of cases, both in laboratory settings and in living organisms. In a study conducted by Shukla et al. (2005), it was found that the consumption of apigenin by transgenic adenocarcinoma prostate mice had a preventive effect on prostate tumorigenesis. This was achieved by the interference of apigenin with the NF-κB signaling pathway in a prostate mouse model. Apigenin therapy has been found to have a positive impact on reducing prostate tumor volume and effectively eliminating cancer cells (Shukla et al., 2005). Based on the studies, it has been found that apigenin has the ability to prevent the phosphorylation and degradation of IB by stopping IKK activation. This in turn leads to suppression of NF-κB activation (Lessard et al., 2006). Apigenin did not interfere with the expression of NF-κB in lung cancer A549 cell line. However, it can also hinder the movement of NF-kB from the cytoplasm space to the nucleus. This leads to suppression of genes that prevent apoptosis. Apigenin also prevents the degradation of IB in lung cancer, ensuring that IB remains bound to the NF-κB heterodimer (Chen et al., 2016). Apigenin therapy was found to effectively inhibit NF-κB nuclear translocation and AKT activation, while also modulating MAPK signaling in preclinical studies (*in vitro* and *in vivo*) in malignant mesothelioma (Masuelli et al., 2017). According to reports, activation of the PI3K/AKT signaling by the epidermal growth factor receptor (EGFR) can lead to various cellular events, including the activation of IKK, phosphorylation of IκBα on serine 32/36, translocation of NF-κB, and signaling through protein kinase (Ck-2) to activate HER-2. These events ultimately result in transactivation of NF-κB. Apigenin has the ability to block LPS and miR-33 activity, leading to the inhibition of cytokine expression and the suppression of oncogenes inside the nucleus. In this way, apigenin can inhibit the growth and spread of cancer cells via repressing NF-κB pathway.

## Baicalein

Baicalein, a flavonoid extract obtained from the dried root of *Scutellaria baicalensis* Georgi. This natural flavonoid has the ability to block cancer-promoting pathways such as metastasis, angiogenesis, and inflammation, while non-toxic to healthy cells (Gupta et al., 2022). The anticancer activity of baicalein mainly depend on its ability to inhibit many intricate cascades. Baicalein exerts its effects by regulating cell cycle targets (cyclins), as well as on free oxygen species scavenging, MAPK, AKT/mTOR, MMP-2/9 expression, and caspase-3/9 stimulation. This stimulates programmed cell death and suppress the invasion, migration, and advancement of tumors (Tuli et al., 2020). It has been also utilized as an antioxidant, antiviral, antibacterial, anti-inflammatory, anti-allergic agent, and for various other purposes (Dinda et al., 2017). Baicalein exerts its effects through various biological mechanisms, including reduction of cancer cell survival, inflammation, angiogenesis and metastasis, as well as induction of apoptotic cell death and autophagy (Morshed et al., 2023).

The ability of baicalein to inhibit cancer cell growth has been extensively studied in numerous experimental research (Lei et al., 2024). By inducing apoptosis, baicalein showed remarkable growth inhibitory potential against cervical cancer cells (Peng et al., 2015). Furthermore, it effectively represses the growth of human prostate and ovarian cancer cells (Miocinovic et al., 2005; Chen et al., 2013a). In the study by Shehatta et al. (2022), EAC-inoculated mice were administered baicalein at a dose of 50 mg/kg/day for a duration of 3 weeks as part of their *in vivo* tests. Additionally, mice were treated with a combination of baicalein and 5-FU. Their findings revealed that baicalein significantly decreased angiogenesis and inflammation by downregulating the expression of NF-κB, IL-1β and VEGF, while also activating apoptotic pathways through increasing the ratio of pro-apoptotic and anti-apoptotic genes (Shehatta et al., 2022). Notably, the antagonist of kappa-B (IκB) plays a crucial role in controlling NF-κB signaling, and disturbances in this pathway have been associated with different types of malignancies (Morgan et al., 2020). Moreover, the Akt pathway has a substantial impact on tumor formation (He et al., 2018). Administration of baicalein can mitigate these effects by decreasing the levels of p-Akt/p-mTOR, p-IκB and NF-κB, and while simultaneously increasing the expression of IκB (Yan et al., 2018).

## Quercetin

Quercetin is a flavone found in several fruits and vegetables such as peppers, apples, berries, cherries, tea, grapes and wine. Quercetin is a naturally occurring compound that possesses anti-cancer, anti-proliferative, and apoptosis-inducing properties (Salehi et al., 2020). Considering its anticancer properties, it can potentially be used as a therapeutic agent to combat tumors. Research has shown that quercetin has the potential to inhibit the growth of carcinomas (such as lung, colon, prostate and breast) in various organs. The mechanism behind the growth inhibitory potential of quercetin was found to be apoptotic induction and cell cycle arrest of cancer cells (Asgharian et al., 2022).

Numerous *in vitro* studies have confirmed the anti-proliferative effects of quercetin and its impact on the expression level of apoptosis and cell cycle regulatory genes. According to previous research, quercetin has the ability to modify the expression of numerous genes and exert antiproliferative, anti-inflammatory, and antitumor properties via modulation of PI3K/AKT/NF-κB or STAT3 pathway (Almatroodi et al., 2021; Carrillo-Martinez et al., 2024). Quercetin exhibits a favorable impact by stimulating the Nrf2/ARE pathway, which enhances the production of antioxidant enzymes such as superoxide dismutase. Furthermore, quercetin suppresses the activation of genes associated with pro-apoptosis and pro-inflammation that are controlled by NF-κB (Acharya et al., 2010; Ramyaa et al., 2014). The NF-κB transcription factor was found to be constantly active in acute myeloid leukemia blasts, other hematological malignancies, and many solid tumors (Rushworth et al., 2012). Quercetin has a suppressive effect on the proliferation of malignant cells including colorectal cancer, bladder, liver, or ovary (Yuan et al., 2012; Tao et al., 2015). In a recent study, Rubio et al. (2018) shown that when leukemia cells were exposed to 25 µM quercetin for 24 h, it had an impact on their proliferation by modulating the NFκB/Nrf2 pathway. In addition, quercetin reduces the survival rate of prostate cancer cells and promotes apoptosis in colon cancer cells through its interference with the NF-κB signaling pathway (Zhang et al., 2015a; Ward et al., 2018). In a recent study, Sahyon et al. (2022) demonstrated the potential of a combination therapy involving quercetin and sulfamethoxazole to exert anticancer effects. They found that this combination induced apoptosis via regulation of caspase-3 and NF-κB genes, and demonstrated selective toxicity against cancer cells (Sahyon et al., 2022). In addition, the authors replicated these findings in live mice that had been inoculated with Ehrlich ascites carcinoma (EAC), serving as animal models. EAC is a type of carcinoma that has the potential to spread within the abdominal area, affecting both the liver and kidneys. As part of their experiment, the researchers administered a combination of sulfamethoxazole and quercetin (200 mg/kg/day) to mice inoculated with EAC for 14 days (Sahyon et al., 2022). These studies clearly indicated the anticancer potential of quercetin via targeting NF-κB pathway in cancer cells.

## Genistein

Genistein is a naturally occurring compound that belongs to the isoflavonoids class of substances. It has the chemical formula C_15_H_10_O_5_. It belongs to the category of phytoestrogens and has been found to inhibit angiogenesis. Genistein is a key secondary metabolite found in *Glycine* max and *Trifolium species*. Legumes are a major source of genistein, but they can also be obtained from other food categories (Rasheed et al., 2022). Several reports have asserted that the presence of genistein in soybeans lowers the chance of developing several cancers, such as prostate, colon, and breast cancer (Sarkar and Li, 2002).

In a study by Pan et al. (2012), genistein significantly inhibited TNBC cell growth. These effects are dependent on the dosage and duration of exposure. MDA-MB-231 cells were exposed to varying concentrations of genistein (5–10–20 µM) for 72 h. The researchers then assessed the impact on apoptosis and the cell cycle, specifically in the G2/M phase. The observed effect was caused by the ability of genistein to inhibit NF-kB through the NOTCH-1 signaling pathway, resulting in modulated expression of Bcl-2 and Bcl-xL (Pan et al., 2012). In their study, Davis et al. (1999) investigated the genistein’s mode of action by using both LNCaP and PC-3 prostate cancer model cell lines. This study demonstrated that genistein effectively inhibited the nuclear translocation of NF-κB and decreased NF-κB DNA binding. This, in turn, triggers the stimulation of the apoptotic pathway (Davis et al., 1999). After pre-treatment with 50 µM genistein for 48 h, the prostate cancer model cells were able to inhibit NF-κB activation, even when inducers such as H_2_O_2_ and TNF-α were added. Genistein has been found to inhibit NF-kB-DNA binding by interfering with the nuclear translocation of the p50 and p65 subunits. In addition, prostate cancer model cells pretreated with genistein showed a decrease in phosphorylated IκBα. This decrease resulted in the formation of an unphosphorylated IκBα-NF-κB complex, which in turn allowed NF-κB to remain in the cytoplasm and prevented its translocation into the nucleus. The inhibition of NF-κB triggers a pro-apoptotic response. In addition, studies have demonstrated that genistein triggers caspase-3 activation and promotes apoptosis in LNCaP and PC-3 prostate cancer cell lines (Davis et al., 1999). Previous studies have uncovered the efficacy of genistein in combating bladder cancer in preclinical models. It was found that genistein has the capability to hinder the proliferation of 253J B-V human bladder cancer cells. Additionally, genistein halted the cell cycle in the G2-M phase. Genistein has been shown to promote apoptosis and downregulate the NF-κB pathway. Genistein’s antitumor potential was demonstrated in an *in vivo* orthotopic tumor mouse model, where it effectively reduced tumor volume by inducing apoptosis in tumor cells and inhibiting angiogenesis (Singh et al., 2006). Xie et al. (2016)’s research findings showed that 20 µM genistein halted the growth of multiple myeloma cells and causes them to undergo apoptosis via blocking the NF-κB signaling pathway. In a recent study, Ozturk et al. discovered that genistein plays a crucial role in inducing apoptosis in thyroid cancer cells by inhibiting the NF-κB pathway (Ozturk et al., 2018).

## Luteolin

Luteolin is a natural phytocompound (flavonoid) that can be obtained from different variety of plant species. It is abundant in various fruits and vegetables, including celery, sweet bell peppers, chrysanthemum (flowers), carrots, broccoli, onion (leaves), and parsley. Luteolin functions as both an antioxidant and a pro-oxidant biochemically, and it demonstrates a variety of biological benefits including anti-inflammatory, anti-allergy, and anti-cancer properties. Furthermore, it is possible that the biological properties of luteolin are functionally interrelated; for instance, the anti-inflammatory action of luteolin may be related to its anticancer properties (Imran et al., 2019). *In vitro* experimental findings have demonstrated the significant impact of MMP-9 and IL-8 on the progression of breast cancer. In breast cancer MCF-7 cells exposed with 12-O-tetradecanoylphorbol-13-acetate (TPA), luteolin inhibited the production of IL-8 and the activation of MMP-9 (Jeon et al., 2015). Furthermore, it suppressed mRNA level by inhibiting the MAPK signaling cascade and reducing the levels of nuclear NF-κB and AP-1. In addition, luteolin prevented the phosphorylation of ERK ½ generated by TPA and repressed the ERK 1/2 pathways that lead to the production of IL-8 and MMP-9 (Jeon et al., 2015). Luteolin has demonstrated efficacy in the treatment of pancreatic cancer by promoting apoptosis, halting the cell cycle, and inhibiting phosphorylation of protein and its signaling. Due to its potentially beneficial anticancer properties, luteolin suppresses the K-Ras/NF-κB/GSK-3β signaling pathway, causing pancreatic cancer cells to undergo apoptosis *in vivo*. This process is followed by the release of cytochrome c, caspase 3 stimulation, and a decrease in the Bcl-2/Bax ratio (Johnson et al., 2015). Luteolin pretreatment of A549 cells inhibited the morphological changes and reduction in E-cadherin level induced by TGF-β1. In addition, activation of the PI3K-Akt-IκBa-NF-κB-Snail pathway, which causes a decrease in E-cadherin levels generated by TGF-β1, was reduced when luteolin was administered as a pretreatment (Chen et al., 2013b). The introduction of an MKP-1 mutant that was resistant to degradation greatly reduced the activation of JNK and the cytotoxic effects induced by luteolin. This suggests that suppression of the JNK suppressor MKP-1 plays a crucial role in luteolin-induced cell death in lung cancer cells. Similarly, luteolin induced cell death and reduced tumor growth in a xenograft model of HepG2 hepatocarcinoma cells. Moreover, it suppressed the DNA-binding ability of NF-κB and ROS generation, which inside the cells mediate the AMPK-NF-κB signaling pathway (Hwang et al., 2011). Huang et al. (2019)’s study demonstrated that treatment with luteolin (10 and 30 µM) for 24 h inhibited the invasion and stimulate cell cycle arrest of breast cancer cell lines. This was achieved by blocking the NF-κB pathway, which in turn decreased the expression levels of c-Myc and human telomerase reverse transcriptase (hTERT) (Huang et al., 2019).

## EGCG

Green tea is a common beverage that is popular among people all over the world. It is believed by many to have possible health advantages, such as the ability to prevent certain diseases like cancer. Green tea is rich in catechins, a specific type of polyphenol. Epigallocatechin-gallate (EGCG) is the most prevalent and biologically potent component among them. EGCG’s anticancer activities have been shown in numerous *in vitro*, *in vivo*, and clinical studies (Lecumberri et al., 2013). Green tea extract and EGCG have been shown to have anticancer properties. These mechanisms include activation of the detoxification system and stimulation of antioxidant activity, modification of the cell cycle, inhibition of the receptor protein kinase (RPK) and mitogen-activated protein kinase (MAPK) pathways, prevention of clonal expansion of the population of tumor-initiating stem cells, and generation of epigenetic modifications in gene expression (Lambert and Elias, 2010; Gupta et al., 2004; Shimizu et al., 2011; Mokra et al., 2022; Lee et al., 2005). The research conducted by Sen and colleagues showed that exposing MDA-MB-231 cells to a concentration of 20 mmol/L EGCG for a duration of 48 h resulted in a significant decline in the protein expression of NF-κB, in comparison to the control group. EMSA analysis demonstrated that treatment with EGCG significantly suppressed the DNA-binding ability of NF-κB and AP-1 to the nuclear protein compared to the untreated control group (Sen et al., 2010).

EGCG treatment effectively suppressed cancer cell proliferation by inducing apoptosis without causing noticeable harm to normal cells. EGCG effectively repressed the migration and invasion of SW780 cells at concentrations ranging from 25 μM to 100 μM. Western blot analysis indicated that EGCG triggered apoptosis in bladder cancer cells by caspase stimulation and modulated expression of Bax, Bcl-2, and PARP level. Additionally, an animal investigation showed that EGCG (100 mg/kg intraperitoneal injection) dramatically reduced tumor weight (68.4%) and tumor volume (58.4%) in mice with SW780 tumors. Moreover, in both the tumor model and SW780 cells, EGCG reduced the mRNA and protein expression of NF-κB and MMP-9. Cell migration and proliferation were not significantly affected by EGCG when NF-κB was suppressed (Luo et al., 2017). After administering EGCG, there was a notable reduction in tumor weight in comparison to control. In addition, treatment with EGCG caused a noticeable reduction in the number of capillaries and downregulated expression of VEGF in the tumor. EGCG at a concentration of 50 μg/mL effectively prevented the HIF-1α and NF-κB stimulation, along with the expression of VEGF in E0771 cells in comparison to control. This finding provides evidence to the idea that EGCG, a well-known catechin found in green tea, inhibits breast cancer tumor development, migration, angiogenesis, and proliferation by directly targeting tumor cells and the surrounding tissue. The process is facilitated by inhibiting the production of VEFG and activation of HIF-1α and NF-κB (Gu et al., 2013). EGCG exerts a negative influence on the expression of many transcription factors, such as Sp1, AP-1, and NF-κB, thereby inhibiting cancer development (Shimizu and Weinstein, 2005). Tian and colleagues recently discovered that a concentration of 20 µM EGCG can effectively enhance the sensitivity of ovarian cancer cells to cisplatin. This is achieved by modifying the c-Myb-mediated NF-κB/STAT3 signaling pathway (Tian et al., 2019). Moreover, Suhail et al. (2023), found that EGCG significantly retarded the cell growth, induced apoptosis, and repressed NF-κB activity in pancreatic cancer. EGCG also downregulated the expression of NF-κB target genes including BCL-2, MMP2, MMP9, and cMyc.

## Diosmetin

Diosmetin is a bioflavonoid that is primarily found in sweet oranges and lemons. It possesses multiple biological attributes including antimicrobial, antitumor, anti-inflammatory, antioxidant, and estrogenic effects (Saini et al., 2022). Multiple studies have exhibited the potential of diosmetin to inhibit cell growth, prevent metastasis, and induce cell death in breast cancer and hepatocellular carcinoma cells (Raza et al., 2024). In addition, diosmetin has been found to exhibit cytotoxic effects on HT-29, Colo205, and Caco-2 cells, indicating its potential as an anti-tumorigenic agent for human colorectal cancer (Zhang et al., 2019; Liu F. Y. et al., 2023). Koosha et al. (2019) have shown that diosmetin suppresses cell proliferation and triggers apoptotic pathways in human colorectal cancer by inhibiting the bone morphogenetic protein (BMP) and NF-κB signaling. It was demonstrated that in cells treated with diosmetin at a concentration of 3.58 μg/mL for 48 h, a significant augmented expression of TNF-α receptors were observed. This, subsequently, triggers the extrinsic pathway of apoptosis. It is widely recognized that TNF-α can induce NF-κB (Poma, 2020). The researchers found that even if the TNF-α receptors were increased, the movement of NF-κB was blocked due to the excessive production of IκB-a. In addition, the decrease in protein expression of survivin provides further evidence of NF-κB inhibition. Therefore, diosmetin induces apoptosis by interacting with apoptotic factors and inhibiting NF-κB (Qiao et al., 2016; Zhang and Luo, 2023).

## Nobiletin

Nobiletin, also known as 5,6,7,8,3′,4′-hexamethoxyflavone, is a naturally occurring flavonoid that is extracted exclusively from citrus peel. It has been found to possess a favorable safety record and a number of pharmacological properties, such as anti-inflammatory effects, antitumor, and neuroprotective capabilities (Cheng et al., 2024). The chemopreventive properties of nobiletin have been previously examined in several types of cancer (Moazamiyanfar et al., 2023). Nobiletin effectively suppressed the activity and protein levels of MMP-2 enzyme in HONE-1 and NPC-BM cells. Nobiletin markedly decreased the attachment of NF-κB and AP-1 to specific sections of MMP-2 gene responsible for initiating its transcription. Nobiletin significantly decreased the number of lung metastatic nodules in mice treated with HONE-1 cells (Chien et al., 2015). Nobiletin reduced the CXCR4 expression in MDA-MB-231 cancer cells. The CXCR4 promoter contains several NF-κB binding sites. Thus, suppressed constitutive activation of NF-κB by nobiletin leads to the reduction of CXCR4 in breast cancer cells (Baek et al., 2012).

Nobiletin has been shown to deactivate FAK and PI3K/Akt in human gastric cancer AGS cells, thereby reducing angiogenesis. Nobiletin decreased the concentrations of phosphorylated Akt and matrix metalloproteinase-2/9 in AGS cancer cells that were transfected with Akt. Nobiletin significantly reduces the amount of nuclear NF-κB and its interaction with NF-κB response elements (Lee et al., 2011). This indicates that nobiletin has the ability to hinder or prevent the development of cancer through the FAK/Akt/NFκB/MMP-2/9 pathway. Nobiletin, at a dose of 100 μM, decreased the expression of MMP-2/9 in osteosarcoma cell lines (U2OS and HOS cells) by inhibiting JNK, ERK, and NF-κB. In addition, nobiletin suppressed the movement, infiltration, and motility of the osteosarcoma cells. These findings indicated that NF-κB may play a role in controlling the invasion of cancer cells (Cheng et al., 2016).

A recent investigation conducted on prostate cancer cells (PC-3 and DU-145) revealed that nobiletin effectively prevents the movement of NF-κB into the cell nucleus. Nobiletin suppressed the AKT phosphorylation and decreased the HIF-1α and VEGFA expression (Chen et al., 2014). A study with comparable findings confirmed that the suppression of VEGFA in ovarian epithelial cells occurs via the AKT-NF-κB-HIF-1α pathway. Nobiletin significantly suppressed the proliferation of tumors originating from subcutaneously administered A2780/CP70 cancer cells in rodent models (Chen et al., 2015). Nobiletin effectively suppressed the survival of human pancreatic cancer cells by causing G0/G1 arrest and declining the cyclin D1 and CDK4 expression. In addition, nobiletin demonstrated dose-dependent suppression of NF-κB, suggesting its potential involvement in the anti-metastatic properties of nobiletin (Jiang F. et al., 2020). Nobiletin causes an increase in nuclear concentrations of NF-κB and initiates programmed cell death and inflammatory cell death. Transfection with miR-200b mimics resulted in noticeable upregulation of NLRP3 or GSDMD expression in cancer cells. Nobiletin significantly enhanced the pyroptotic effects of the miR-200b mimics. Nobiletin and miR-200b mimics showed substantial pyroptotic effects on breast cancer cells. However, the combination of nobiletin and the miR-200b mimic resulted in even more dramatic effects. miR-200b directly targets JAZF1 (juxtaposed with another zinc finger protein 1). The expression of JAZF1 was dramatically suppressed in cancer cells transfected with miR-200b mimics, whereas the levels of JAZF1 were significantly increased in the presence of miR-200b inhibitors. The use of miR-200b inhibitors resulted in deactivation of the NF-κB pathway in BT549 cancer cells treated with nobiletin. However, these effects were more significant in cancer cells overexpressed JAZF1 (Wang et al., 2021b).

Nobiletin exerts anticancerous potential in breast cancer cells mainly by reducing the activation of NF-κB and its control over nuclear receptors, such as retinoid-related orphan receptors (RORs). Remarkably, studies have demonstrated that nobiletin enhances the binding of ROR to response elements in the IκBα promoter and effectively inhibits the translocation of p65 to the nucleus. In contrast, an increase in p65 expression nullified the therapeutic effects of nobiletin against triple-negative breast cancer (TNBC). Suppressing NF-κB expression to enhance the circadian cycle of nobiletin can serve as a unique approach for the chemoprevention and therapy of this highly lethal and aggressive type of breast cancer. The combination of docetaxel and nobiletin effectively caused tumor xenografts to shrink in mice that had been orthotopically injected with MDA-MB-231 cancer cells. The antitumor effects of nobiletin were also examined in DB7 cells induced xenograft mice model with a fully functional immune system. The average size of the tumors in nobiletin-treated mouse models was significantly reduced. In addition, the levels of TNF-α in both the plasma and tumor were dramatically reduced by nobiletin (Kim et al., 2022).

## Wogonin

Wogonin is a bioactive compound derived from the *S. baicalensis* and has the chemical formula 5,7-dihydroxy-8-methoxyflavon. It can also be found as wogonoside in four different species of the Scutellaria genus (Huynh et al., 2020). Several preclinical studies have demonstrated the potential of wogonin in inhibiting tumor growth through mechanisms such as cell cycle arrest, induction of cell death, and prevention of metastasis. Recent studies have demonstrated the antitumor properties of wogonin, a flavone isolated from *S. baicalensis* Georgi. It has been shown to block the activity of NF-κB, a protein involved in cancer development. Specifically, the results shown by Liu et al. (2016) showed that human chemoresistant myelogenous leukemia cells can be naturally sensitized by inhibiting Nrf2 via NF-κB signaling when treated with increasing doses of wogonin.

## Icariin

Icariin is a phytochemical that belongs to the group of flavonoid glycosides. It possesses a range of pharmacological properties and serves as the primary active component in the extract of Herba Epimedium (He et al., 2020). Recent advancements in modern pharmacological research have revealed significant advancements in the bioactivity of icariin in several areas such as cancer, immunology, neurological and cardiovascular system (Verma et al., 2022). Multiple studies have demonstrated that icariin can hinder the growth of tumor cells by controlling many pathways. These pathways include restraining the proliferation and migration of tumor cells, prompting cell differentiation and death, regulating autophagy, and obstructing the cell cycle. In addition, research data suggest that the activity of icariin and its metabolites in fighting cancer is connected to various transcription factors, protein kinases, and growth factors including NF-κB (Liu J. et al., 2023).

Song et al. (2020) discovered that icariin effectively suppressed the cell proliferation and promoted apoptosis through the mitochondrial pathway in breast cancer cells. Furthermore, it suppressed the invasion and migration of MDA-MB-231 cells via the SIRT6/NF-κB/EMT signaling pathway. Zhang et al. (2014), discovered that icariin can effectively increase the susceptibility of colorectal cancer cells to radiotherapy and is linked to the suppression of NF-κB activity. Currently, 5-fluorouracil (5-FU) is extensively utilized in clinical settings as a primary chemotherapy medication for cancer treatment (Vodenkova et al., 2020). Nevertheless, the ultimate effectiveness of chemotherapy medications is suboptimal because of the development of drug resistance in patients with cancer. Recent research has shown that icariin can suppress the proliferation of tumor cells by downregulating NF-κB activity. Additionally, it can stimulate its effectiveness of 5-FU in fighting against colorectal cancer. Li et al. (2014) examined that the combination of icariin and arsenic trioxide (ATO) effectively repressed the growth and promoted apoptosis of human hepatocellular carcinoma (HCC). This combination treatment showed superior effectiveness compared with ATO alone. The antitumor efficacy of icariin and its ability to augment the antitumor effects of ATO are associated with the production of intracellular ROS and suppression of NF-κB activity (Li et al., 2014). El-Shitany and Eid (2019) examined the anti-inflammatory effects of icariin by studying its impact on both NF-κB and HO-1/Nrf2 pathways. In a study conducted on rats with acute inflammation, icariin pre-treatment resulted in decreased expression of inflammatory cytokines, reduced expression of the COX-2 gene, and inhibited activation of NF-κB (El-Shitany and Eid, 2019).

## Astragalin

Astragalin is a flavonoid found in various plants such as persimmon leaves, green tea seeds, horseradish tree leaves, lotus leaf, *Chinese rose, Cuscuta chinensis, Morus alba* L., and *Thesium chinense*. It is also known as kaempferol 3-Oβ-D-glucopyranoside and has the chemical formula C_21_H_20_O_11_. Due to its exceptional bioactivities and potential therapeutic properties for a wide range of disorders, astragalin has become a research hotspot in recent years (Riaz et al., 2018). A growing body of research suggested that astragalin may inhibit several malignancies, including those affecting the digestive tract, skin, breast, lungs, liver, and kidneys. It is noteworthy that these results partially revealed the underlying mechanisms involved in modifying the NF-κB, PI3K/AKT, and JAK/STAT pathways to induce apoptosis and inhibit the development of tumor cells (Chen et al., 2023).

A study found that astragalin inhibits gastric cancer cell proliferation, invasion, and migration by reducing the levels of tumor-associated antigens and gastric epithelial glycoprotein MUC1. Radziejewska et al. (2022) found that astragalin inhibited the production of Tn, sialyl Tn, and T antigens by reducing the activity of various glycosyltransferases in AGS cells. Additionally, astragalin reduced the amount of NF-κB, indicating that the NF-κB pathway may play an important part in preventing the glycosylation process (Radziejewska et al., 2022).

Furthermore, a recent study has demonstrated that astragalin has the ability to inhibit the NF-κB pathway, hence preventing the formation of colon cancer cells in nude mice. Astragalin administration reduces the size of colon tumors in living organisms and decreases the phosphorylation of IKKα and NF-κB. Moreover, experiments conducted in a controlled environment outside a living organism showed that astragalin inhibited the growth and movement of HCT116 cells by reducing the expression of MMP-2 and MMP-9. It also halted the cell cycle progression by decreasing the levels of crucial cyclins and CDKs, and increasing the levels of P21 and P27. Additionally, astragalin triggered cell death by increasing the levels of caspases, P53, and Bax and decreasing the levels of cleaved caspase-3 and Bcl-2. The fact that astragalin reduced the levels of p-NF-κB, p-IκBα, p65, TNF-α, and IL-6 suggests that astragalin may target NF-κB signaling as a strategy for colon cancer treatment (Yang et al., 2021).

Chen et al. (2017) found that astragalin suppressed the size and weight of tumors in xenograft nude mice, enhanced the number of apoptotic cells, and blocked clonogenic cell proliferation. Astragalin therapy resulted in the upregulation of caspases, PARP, Bax, Bad, and Fas level, and downregulation of Bcl-xl and Bcl-2. Furthermore, the MAPK pathway was deactivated and LPS was inhibited by astragalin application, and TNF-α stimulated the activation of the NF-κB pathway in A549 cells by downregulating the phosphorylation level of p-PI3K/Akt, p38, ERK, IκBα, and IKK-β and upregulating p-JNK. It is worth noting that the MEK and PI3K/Akt inhibitors showed similar effects as astragalin, while the caspase antagonist counteracted the pro-apoptotic effect of astragalin (Chen et al., 2017).

## Oroxylin A

Oroxylin A is a naturally occurring molecule that belongs to the class of O-methylated flavones. It is mostly found in the medicinal plant *Oroxylum indicum*, which has been widely utilized in Ayurveda. The substance is also derived from the dried roots of plants that are significant to traditional Chinese medicine (TCM), including *S. baicalensis*, *Scutellaria lateriflora, Anchietea pyrifolia*, and *Aster himalaicus*. This naturally occurring compound has great medicinal potential (Lu et al., 2016). Multiple preclinical investigations have demonstrated that OA has therapeutic properties in a diverse range of malignancies, such as breast, colon, cervix, leukemia, lung, pancreatic, ovary, and skin cancers (Tuli et al., 2023).

Sun et al. (2019b) proposed that oroxylin A compound derived from the *O. indicum* and *Scutellariae radix* and, may be a potential therapeutic agent for breast cancer treatment. According to their research, a 24-h treatment with a concentration of 20 µM of Oroxylin A effectively hinder cell sustainability, migration, invasion, and epithelial-mesenchymal transition (EMT) by deactivating the NF-κB pathway in breast cancer cells. Further investigation showed that OA, extracted from *S. baicalensis*, hindered the growth of HCT116 cells by decreasing NF-κB signaling (Yao et al., 2014).

Another study showed that oroxylin A markedly inhibited the cell proliferation and differentiation of acute myeloid leukemia (AML) cells generated by TNF-α. In NB4 and HL-60 resistant cells, oroxylin A decreased the expression of truncated retinoid X receptor alpha (tRXRα) to suppress the PI3K/Akt pathway (Li et al., 2018). Additionally, this study showed that oroxylin A combined with tumor necrosis factor alpha (TNFα) decreased the number of AML cells and increased the lifespan of AML xenograft models (Li et al., 2018).

Oroxylin A was demonstrated to inhibit the production of Tregs produced by H460 lung cancer cells when they were cultured with peripheral blood mononuclear cells via lowering TGF-β1 secretion (Shen et al., 2017). Oroxylin A has also been shown to decrease NF-κB signaling in H460 cells. Zhao et al. (2018) showed that oroxylin A decreased the movement and formation of blood vessel-like structures in endothelial cells by reducing the phosphorylation level of important signaling proteins such as pAkt, pERK, pVEGFR2, p38, pIKK, pIκB and increasing the levels of Ras and E-cadherin (Zhao et al., 2018). Oroxylin A also suppressed tumor growth and angiogenesis in A549 xenograft mice by downregulating pVEGFR2 and inducing Ras and cadherin expression (Zhao et al., 2018).

## Other flavonoids

In addition to these compounds, the anticancer properties of some other flavonoids, such as hesperetin and chrysin, have also been reported by targeting the NF-κB pathway. Kong et al. (2020b) reported that hesperitin enhanced sensitivity of lung cancer cells towards cisplatin. Additionally, hesperetin pretreatment in drug-resistant A549 cells suppressed NF-κB (p65) activity and translocation to the nucleus. In another study, Cheng et al. showed that hesperetin inhibited the growth and metastasis of glioblastoma GL261 cells by downregulating the expression of Akt, TNF-α, NF-κB, MMP-2, MMP-9, N-cadherin, and vimentin (Cheng et al., 2022). Another flavonoid, chrysin, has also been found to inhibit the NF-κB pathway in lung cancer cells. A study done by Kasala et al. (2016), found that chrysin treatment downregulated the expression of COX-2, NF-κB and PCNA in benzo(a)pyrene induced lung cancer mice model. Similarly, Wu et al. (2018), also showed that chrysin repressed the phosphorylation levels of IκB and IKKβ and the nuclear level of p65 in lung cancer A549 cells.

## Conclusion and future perspectives

Numerous processes, including metastasis, angiogenesis, tumor progression, and drug resistance, are mediated by NF-κB pathway. Genetic changes that regulate NF-κB activation may augment NF-κB activity, and significantly affect cancer development. Molecular targets that aim to inhibit NF-κB activity have gained significant attention in the fight against cancer and its treatments. Phytochemicals are valuable resources that can be used as potential drugs. Plants are a rich source of complex molecules that can exhibit highly specific or broad-ranging activities. The therapeutic potential and intricacy of NF-κB-affecting drugs are enhanced by their ability to control other pathways including PI3K, AKT, MAPK, and p53. Recent studies have discussed the role of flavonoids in regulating the NF-κB signaling pathway. Due to these reasons, the scientific community is currently directing its attention towards flavonoids found in various plants as a potential alternative approach to combat or prevent cancer. Based on the facts documented in this review, it is suggested that flavonoids have the potential to prevent cancer by modulating the NF-κB pathway either directly or indirectly. The application of flavonoids for the management of cancer is a complex field with great potential for clinical advancement. An in-depth understanding of the flavonoid structure, metabolism, and molecular functions may facilitate the use of flavonoids as alternative medications. A comprehensive understanding of the relationship between various functional groups in flavonoid structures and their influence on molecular mechanisms is essential for further development and modification of flavonoid structures to enhance their therapeutic efficacy. This knowledge will facilitate the development of enhanced alternative approaches for the management of various carcinomas. Although flavonoids have been shown to have extraordinary and advantageous pharmacological effects, there are still many issues surrounding their effects, particularly in light of the scarcity of epidemiological evidence and limited bioavailability. Hence, it is imperative to explore various techniques to enhance the bioavailability of flavonoids and their anticancer efficacy.
